# Urinary Incontinence (UI) in Saudi Female Population: Prevalence, Risk Factors, and Impact on Quality of Life

**DOI:** 10.3390/healthcare12232340

**Published:** 2024-11-23

**Authors:** Abrar H. Alharbi, Ruba H. Almasry, Basim Alsaywid, Ahlam Kaleemullah, Ahmed T. Khodri, Farss S. Hariri, Salahadin H. Lamy, Talah O. Almaddah, Miltiadis D. Lytras

**Affiliations:** 1 Batterjee Medical College, Jeddah 21442, Saudi Arabia; rubahalm@gmail.com (R.H.A.); ahlamkaleem68@gmail.com (A.K.); dr.farsshariri@gmail.com (F.S.H.); talah_almaddah@outlook.com (T.O.A.); 2 Education and Research Skills Directory, Saudi National Institute of Health, Riyadh 12382, Saudi Arabia; drbasim@yahoo.com; 3 Pediatric Urology Section, Department of Surgery, College of Medicine, King Saud University Medical City, King Saud University, Riyadh 11421, Saudi Arabia; 4 Urology Department, Dr. Soliman Fakeeh Hospital, Riyadh 13325, Saudi Arabia; 5 Ibn Sina National College, Jeddah 22421, Saudi Arabia; ahmed.khodri03@gmail.com; 6 King Abdulaziz Medical City, Jeddah 22384, Saudi Arabia; drsalahadin@outlook.com; 7 Effat College of Engineering, Effat University, Jeddah 21478, Saudi Arabia

**Keywords:** obesity, pregnancy, quality of life, risk factors, Saudi females, stress incontinence, urgency incontinence, urinary leakage, urology, woman’s health

## Abstract

Background: Urinary incontinence (UI) is a prevalent health concern among women globally. However, its prevalence, associated risk factors, and impact on quality of life among Saudi women remain underexplored. Objective: This study aimed to assess the prevalence of UI, identify its risk factors, and understand its impact on the quality of life among Saudi women. Methods: In this cross-sectional study, we surveyed Saudi women aged between 18 and 50 years. Participants were recruited from shopping malls to reflect a diverse demographic. The questionnaire included detailed questions about the participants’ experiences with urinary incontinence, their lifestyle and health-related risk factors, and the impact of the condition on various aspects of their daily lives. Results: The study found a 32.4% prevalence of urinary incontinence (UI) among 516 women. Risk factors included age, marital status, BMI, childbirth, and vaginal surgery. UI prevalence increased with age and was more common in married women, women who had children, and those with vaginal surgery history. However, only 29.3% sought medical advice, and 55.2% reported no improvement after consultation. Urinary incontinence impacted respondents’ lives in several ways with 38.9% reporting limitations in social activities, approximately 50% experiencing some degree of impact on household tasks, and about 19.4% facing significant or extreme impact on job or daily activities. Conclusions: UI has a substantial prevalence among Saudi women and significantly affects their quality of life. The study underscores the need for increased awareness, routine screening, and timely medical consultation for the effective management of UI.

## 1. Introduction

Urinary incontinence is a condition marked by involuntary urine leakage [1]. This may occur due to normal physiological changes such as pregnancy, menopause, and aging or due to pathological conditions such as diabetes, neurological disorders, an enlarged prostate, urinary tract infections (UTIs), or constipation [2]. Potential risk factors for urinary incontinence include age, smoking status, being overweight, having a family history of the condition, being female, and suffering from a neurological disorder [3].

The most prevalent type of urinary incontinence is stress urinary incontinence (SUI), which is triggered when pressure is exerted on weak bladder muscles during actions such as sneezing, coughing, laughing, and exercising. The second most common type is urgency incontinence or overactive bladder (OAB). Individuals suffering from this type of injury experience a sudden urge to urinate before the bladder is fully filled. Another type is overflow incontinence, which occurs when the volume of urine produced surpasses the bladder’s capacity, when the patient has a full bladder that cannot be emptied due to blockage, or when the bladder muscles cannot contract correctly. Some patients may experience SUI and OAB simultaneously, which is a condition referred to as mixed incontinence [1,4].

The diagnosis of urinary incontinence requires reviewing the patient’s medical history, conducting a physical examination, measuring the postvoid residual urine volume, and ordering a urinalysis [4]. The primary goal of the initial evaluation is to identify and address any potential reversible causes and to ascertain whether the patient has any conditions that require referral or specialized tests. Urinary incontinence is also associated with comorbidities such as urinary tract infections, constipation, and depression among both men and women [5]. Prior studies on urinary incontinence in Saudi Arabia have highlighted a significant prevalence among various demographics with risk factors including age, obesity, and childbirth history being commonly identified. These studies also underline the profound impact urinary incontinence has on quality of life, affecting social interactions and mental health. Despite this, there remain notable knowledge gaps, particularly concerning the nuanced experiences of different types of urinary incontinence and the intersection of cultural attitudes toward seeking treatment.

The objective of this study was to determine the prevalence, types, and risk factors for urinary incontinence and assess its impact on quality of life among healthy Saudi women in 2023.

## 2. Methodology

### 2.1. Study Design and Population

This study was conducted using an observational, analytical, cross-sectional approach. The study included women living in Saudi Arabia aged 18 to 50 who were healthy and without chronic conditions affecting urinary function. Exclusions applied to postmenopausal women, those who were pregnant or postpartum within the past 3 months, and those with medical conditions, surgeries, or medications affecting bladder control. The inclusion of women aged less than 30 in this study was intentional to provide a comprehensive assessment of UI across a broader reproductive age range. While UI prevalence is generally lower in younger women, early onset of UI can occur and may go underreported in this demographic. Eligible participants were invited to participate in the study by completing a self-administered, online, close-ended questionnaire that had been previously validated.

### 2.2. Study Setting

The study was conducted in Jeddah, Saudi Arabia. To ensure diverse samples representing various regions of the city, data were collected from three distinct malls: Red Sea Mall, Yasmin Mall, and Al Andalus Mall. These malls represent the northern, southern, and eastern regions of the city.

### 2.3. Sampling and Data Collection

For our cross-sectional survey evaluating the prevalence and risk factors of urinary incontinence among women in Saudi Arabia, we utilized a non-probability convenience sampling technique. This approach allowed us to efficiently gather data from participants who were easily accessible within our target population. To estimate our sample size, we considered an anticipated prevalence rate from previous studies, aiming for a confidence level of 95% and a margin of error of 5%. This calculation guided us to a final sample size of approximately 400 participants.

The primary method of data collection involved distributing a self-administered, structured, online, questionnaire with close-ended questions. This questionnaire, designed by a Saudi research team (Alshenqeti et al., 2022) [6], aimed to estimate the prevalence and risk factors for urinary incontinence as well as its impact on quality of life. The survey included demographic information, medical history, gynecological information for women, and information on lifestyle factors such as smoking. The questionnaire also included questions assessing the prevalence of urinary incontinence among participants and questions on risk factors for urinary incontinence and its impact on quality of life, such as the effect of urine leakage on daily activities and night-time sleep. The quality of life was assessed using The King’s Health Questionnaire (KHQ), which is a valid and reliable tool to measure disease-specific health-related quality of life. The tool was previously tested and applied to the Saudi population [7,8].

### 2.4. Statistical Analysis

Statistical analysis was performed using the SPSS software package for Statistical Analysis (IBM Corp. Released 2023. IBM SPSS Statistics for Mac, Version 29.0.2.0 Armonk, NY, USA: IBM Corp). The normality of continuous variables was assessed using histograms and the Shapiro test. All continuous variables in our study were skewed, so they were summarized using the median and interquartile range. Categorical variables are represented using frequency tables and percentages. The potential association with incontinence was investigated using the chi-square test with the significance level for the *p* value set at 0.05. Subgroup descriptive analyses were conducted to examine the types and quality of life among participants who reported incontinence.

### 2.5. Ethical Considerations

Ethical considerations were paramount in this study. Each participant was informed about the study objectives to obtain informed verbal consent. The research team assured the confidentiality of all participants, and all collected data were securely stored and used only for research purposes. The study received approval from the Institutional Review Board (IRB).

## 3. Results

The study sample consisted of 516 women aged 18 to 50 years with a 32.4% overall prevalence of urinary incontinence. The majority (58.1%) were 18–30 years old, while 16.7% were 31–40 years old, and 25.2% were 41–50 years old. Most participants (58.9%) were single, and 84.5% (436) were Saudi nationals. In terms of education, 68.8% held a bachelor’s degree, 20% completed high school, 8.5% had higher education, and 2.7% had intermediate or less education. Regarding employment status, 55.8% were unemployed. The body mass index (BMI) distribution showed that 47.1% had normal weight, 28.7% were overweight, 13% were obese, and 11.2% were underweight. Only 16.1% of the participants were smokers. This diverse sample provided a comprehensive representation of the Saudi female population for the study of urinary incontinence. The detailed results are shown in Table 1.

Regarding marital status, 58.9% were single with a lower prevalence of incontinence compared to married women (25.7% vs. 42%). This finding was statistically significant. The *p* value was less than 0.001. Regarding body mass index (BMI), approximately half (47.1%) had a normal BMI, and only 11.2% were underweight. BMI was significantly associated with the incidence of incontinence reported by the women. The sociodemographic details are shown in Table 1.

Only 3.7% of the participating women were pregnant. While not statistically significant, incontinence was reported by 42.1% of pregnant women and 32% of nonpregnant women. Compared with women who had never conceived, women who had previously conceived were reported to have a prevalence of incontinence of 39.5% (46.1% vs. 23.4%, *p* value < 0.001). In terms of vaginal deliveries, 33.5% of respondents had a history of vaginal delivery, and among these respondents, 48.2% reported urinary incontinence, while 24.2% of those with no history of vaginal delivery reported urinary incontinence. This association was statistically significant (*p* value < 0.001). Furthermore, vaginal gynecologic surgery also showed a significant association with incontinence. Almost half (47.2%) of those who underwent vaginal gynecologic surgery reported urinary incontinence, whereas 30.7% of those with no history of vaginal gynecologic surgery reported urinary incontinence. This association was also found to be statistically significant (*p* = 0.020). Of the respondents, 17.1% had a history of caesarean section (CS), but it was not significantly associated with incontinence. The detailed results are shown in Table 2.

Of the women who reported urinary incontinence, 52.9% had experienced it in the past month. The majority (62.0%) described the amount of leaked urine as a few drops. The leakage frequency in the past week varied with 50.7% experiencing it less than once weekly and 17.9% experiencing it once weekly. Most women (71.9%) reported leakage during physical movements such as coughing, laughing, and sneezing, which is indicative of stress incontinence. Over half (55.1%) reported a sudden and intense need to urinate, suggesting urgency incontinence. Additionally, 39.5% reported a combination of stress and urge incontinence, constituting mixed incontinence. Concerning medical consultation, only 29.3% of women had sought medical advice for their urinary incontinence problem. Of these, a significant number (55.2%) reported that the problem was not solved. More details are provided in Table 3.

Urinary incontinence significantly affected various aspects of the respondents’ lives. Approximately 38.9% of the women reported that urinary incontinence limited their social activities. Approximately half of the participants reported some degree of impact on their household tasks. Similarly, a notable portion of respondents reported an impact on their job or daily activities with 19.4% reporting either a significant or extreme impact. Concerning relationships with partners, 40.1% reported some degree of impact with 18.3% reporting significant or extreme impact. Last, in terms of sleep, 14.1% reported a significant or extreme impact. These results underscore the considerable influence that urinary incontinence can have on various aspects of life. For more details, refer to Table 4.

Regarding daily water intake, 19.8% of the women drank 6 or more cups. Night-time urination was common among the participants with 61.8% getting up once a night, 24.3% twice, and 13.9% three times. Bedwetting was reported by 19.8% of the women. Approximately 47% of the women used pads as a solution for their condition. A total of 29.1% of women reported that their menstruation cycle worsened. Cold weather seemed to exacerbate the condition for 53.3% of respondents. Physical abuse related to urinary incontinence was reported by 5.3% of the women. Finally, 23.6% of women reported that urinary incontinence started after they experienced anxiety. More details are provided in Table 5.

## 4. Discussion

Urinary incontinence (UI) remains a prevalent health concern for women globally [1,9]. The purpose of this study was to assess the prevalence of urinary incontinence (UI) among Saudi women and its impact on quality of life. Our findings revealed a prevalence of UI of 32.4% with stress incontinence being the most common type. However, compared to findings from previous studies in the region (Thabet et al., 2018; Almutairi et al., 2019), which reported a higher prevalence (44.2% and 41.7%, respectively) [10,11], our findings suggest a relatively lower prevalence. This could be attributed to the difference in the methodological approach used. First, our study recruited participants from shopping malls, potentially reflecting a younger demographic with a lower UI prevalence compared to hospital-based studies that might attract women with existing health problems. Second, the mean age in our study (27 years) was lower than that in previously cited studies (42.5 years), and the minor variations and diagnostic criteria used could also be attributed to the observed differences [11]. Our analysis indicated a UI prevalence of 25% among participants aged 30 years or younger. This finding closely aligns with prior research, which reported a prevalence of 24.8% among women aged 35 years or younger. Although there is a slight difference in the age groups examined, the similarity in prevalence suggests a consistent trend of UI in younger women across different studies [11].

The results suggest several factors that might contribute to UI. One is age, as UI prevalence increased with older age groups. This can be attributed to several age-related physiological changes in the lower urinary tract, including detrusor instability/overactivity, a reduction in functional bladder capacity, an increase in postvoid residual volume, decreased awareness of bladder filling, and reduced bladder emptying efficiency [12]. Another possible reason could be age-related hormonal changes, such as a decrease in estrogen, which decreases the integrity of the pelvic floor muscles [13]. This finding is consistent with findings from a systematic review that reported a significant association between age and UI starting to increase significantly at 36.9 years [14].

This study also revealed an association between marital status and UI prevalence. Compared with single women, married women reported a greater prevalence of UI. Although no previous research has established marriage itself as an independent risk factor for UI, this finding might be explained by the established correlation between age and UI. The married women in this study might have simply belonged to an age group with a higher overall prevalence of UI. These findings support a previous study that identified a similar association between increased risk of UI and age particularly at 40 years and older [15]. Further research is necessary to explore this potential link and investigate whether other factors related to marital status might contribute to UI in women.

A statistically significant association was observed between UI and BMI. It is theorized that excess body weight increases abdominal pressure, which can increase bladder pressure and urethral mobility. This may contribute to stress UI and worsen bladder overactivity [16]. These findings are in accordance with a systematic review of the literature by Subak et al., 2009, which revealed that obesity is a strong, independent risk factor for prevalent and incident UI. There is a clear dose‒response effect of weight on UI with each 5-unit increase in BMI associated with an approximately 20% to 70% increase in the risk of UI [16]. Another meta-epidemiological study involving 16 observational studies revealed that obesity (BMI 30–35 kg/m^2^) significantly increases the risk of urinary incontinence (UI) in middle-aged and elderly women. In fact, overweight and obesity are considered as an independent risk factor stress urinary incontinence (SUI) among middle-aged and elderly women [17].

Furthermore, the study revealed a statistically significant association between UI and childbirth, vaginal delivery, and vaginal surgery, which are all known risk factors for weakening of the pelvic floor muscles. These findings support a well-established association between prior deliveries and urinary incontinence (UI), particularly SUI [18]. This finding aligns with previous studies demonstrating that vaginal delivery is a significant risk factor for pelvic floor disorders, including UI. One study found that compared to caesarean deliveries and nulliparous women, vaginal delivery increased the odds of having SUI by 81% [19]. In line with our findings, another study revealed an association between increased parity and UI during the first year postpartum [20]. This study strengthens this understanding by demonstrating a clear relationship between prior deliveries and UI among Saudi women.

This study also highlights the significant impact of UI on quality of life beyond physical leakage. Overall, 38.9% of women reported limitations in social activities, suggesting potential social isolation. The burden of managing UI also affects household tasks in 50.7% of women and significant impact on work or daily activities in 19.4%, potentially leading to stress and decreased productivity. These findings are similar to the findings of the EPIC study, which showed that UI negatively impacts work productivity across various sectors [21]. These findings are concerning, especially considering the substantial impact on sleep and partner relationships (over 14% and 18% reporting significant or extreme impacts, respectively). This study’s findings on the psychological impact of UI are consistent with Shing et al.’s description of UI as a “silent epidemic” due to social stigma and the emotional distress it can cause [22].

This is a concern echoed in the study by Kim et al. (2004) [1]. This study revealed that overactive bladder (OAB), a condition often comorbid with UI, significantly decreased the enjoyment of sexual activity particularly in OAB patients with additional lower urinary tract symptoms (LUTSs) [23]. These findings underscore the importance of effective UI management strategies. Future research needs to explore the cost-effectiveness of various treatment options, considering not only medical costs but also potential improvements in productivity and quality of life. Additionally, investigating the role of psychological interventions for managing UI-related stress and anxiety could be valuable.

This study used a sizeable sample of 516 Saudi women, which strengthens the generalizability of our findings to the target population. Additionally, our study explored underreported aspects of UI, such as its impact on partner relationships, which offers valuable insights into the broader social consequences of UI. However, the recruitment method could result in potential selection bias. Malls tend to attract younger people who may have a lower prevalence of UI. Additionally, the average age of the study population was younger than that in some previous studies, which could influence the observed UI incidence. These limitations highlight the need for future studies with more diverse recruitment strategies and age-inclusive samples to provide a more comprehensive picture of UI in Saudi women.

## 5. Conclusions

Our study found a 32.4% prevalence of UI with stress incontinence being the most common type. Several factors were found to be associated with UI, including age, BMI, childbirth, and vaginal surgery. In addition to physical leakage, UI significantly impacts quality of life with 40% of women reporting limitations in social activities, impaired household tasks and productivity. These findings highlight the substantial impact of UI on social interaction, daily life, and potentially mental well-being. Effective UI management strategies are crucial for improving overall well-being. Future research should explore various treatment options that have the potential to improve productivity and quality of life. Specifically, investigating behavioral therapy for UI management, alongside the role of psychological interventions in managing UI-related stress and anxiety, could be valuable for further exploration.

## Figures and Tables

**Table 1:** Distribution of urinary incontinence across demographic groups and their associated significance levels.

Variables	Groups	Total	Incontinence		*p* Value
			Yes	No	
Age groups (years)	18–30	300 (58.1%)	75 (25%)	225 (75%)	<0.001
	31–40	86 (16.7%)	31 (36%)	55 (64%)	
	41–50	130 (25.2%)	61 (46.9%)	69 (53.1%)	
Marital status	Single	304 (58.9%)	78 (25.7%)	226 (74.3%)	<0.001
	Married	212 (41.1%)	89 (42%)	123 (58%)	
Nationality	Saudi	436 (84.5%)	140 (32.1%)	296 (67.9%)	0.795
	Non-Saudi	80 (15.5%)	27 (33.8%)	53 (66.3%)	
Educational level	Intermediate or less	14 (2.7%)	8 (57.1%)	6 (42.9%)	0.188
	High school	103 (20%)	30 (29.1%)	73 (70.9%)	
	Bachelor	355 (68.8%)	113 (31.8%)	242 (68.2%)	
	Higher education	44 (8.5%)	16 (36.4%)	28 (63.6%)	
Employment	Employed	228 (44.2%)	71 (31.1%)	157 (68.9%)	0.363
	Unemployed	288 (55.8%)	96 (33.3%)	192 (66.7%)	
BMI groups	Underweight	58 (11.2%)	10 (17.2%)	48 (82.8%)	<0.001
	Normal	243 (47.1%)	64 (26.3%)	179 (73.7%)	
	Overweight	148 (28.7%)	54 (36.5%)	94 (63.5%)	
	Obese	67 (13%)	39 (58.2%)	28 (41.8%)	
Smoking	Yes	83 (16.1%)	27 (32.5%)	56 (67.5%)	1.000
	No	433 (83.9%)	140 (32.3%)	293 (67.7%)	

**Table 2:** Distribution of urinary incontinence across obstetric and gynecological characteristics.

Question	Response	Total	Incontinence		*p* Value
			Yes	No	
Are you currently pregnant?	Yes	19 (3.7%)	8 (42.1%)	11 (57.9%)	0.453
	No	497 (96.3%)	159 (32%)	338 (68%)	
Have you been pregnant before?	Yes	204 (39.5%)	94 (46.1%)	110 (53.9%)	<0.001
	No	312 (60.5%)	73 (23.4%)	239 (76.6%)	
Do you have any history of caesarean section?	Yes	88 (17.1%)	33 (37.5%)	55 (62.5%)	0.263
	No	428 (82.9%)	134 (31.3%)	294 (68.7%)	
Do you have any history of vaginal delivery?	Yes	164 (33.5%)	79 (48.2%)	85 (51.8%)	<0.001
	No	326 (66.5%)	79 (24.2%)	247 (75.8%)	
Do you have any history of abdominal gynecologic surgery?	Yes	100 (19.4%)	35 (35%)	65 (65%)	0.553
	No	416 (80.6%)	132 (31.7%)	284 (68.3%)	
Do you have any history of vaginal gynecologic surgery?	Yes	53 (10.3%)	25 (47.2%)	28 (52.8%)	0.020
	No	463 (89.7%)	142 (30.7%)	321 (69.3%)	

**Table 3:** Characterization of urinary incontinence experiences and pursuit of medical consultation.

Question	Responses	N	%
Have you experienced urinary incontinence in the past month?	Yes	82	52.9%
	No	73	47.1%
How much would you estimate the amount of the leaked urine?	Few drops	93	62.0%
	Small volume	46	30.7%
	High amount	11	7.3%
How frequent was the leakage of urine for the past one week?	Less than once weekly	71	50.7%
	Once weekly	25	17.9%
	More than once weekly	17	12.1%
	Once daily	2	1.4%
	More than once daily	25	17.9%
Have you experienced leakage during coughing, laughing or sneezing?	Yes	120	71.9%
	No	47	28.1%
Have you experienced a sudden and intense need to pass urine and cannot delay going to the toilet?	Yes	92	55.1%
	No	75	44.9%
Have you experienced a combination of stress and urge incontinence?	Yes	66	39.5%
	No	101	60.5%
Have you experienced involuntary total bladder emptying triggered by laughing or giggling?	Yes	88	52.7%
	No	79	47.3%
Have you sought a medical consultation for your urinary incontinence problem?	Yes	49	29.3%
	No	118	70.7%
What was the result of the medical consultation you sought?	No improvement	53	55.2%
	Partial improvement	19	19.8%
	Complete improvement	24	25.0%

**Table 4:** Impact of urinary incontinence on quality of life in areas such as social activities, daily tasks, work, driving, sleep, and emotional states.

Question	Responses	N	%
Does your urinary incontinence limit your social life?	Yes	58	38.9%
	No	91	61.1%
Does your urinary incontinence affect your household tasks such as cleaning, cooking, etc.?	Not at all	74	49.3%
	A little	22	14.7%
	Slightly	22	14.7%
	Moderately	13	8.7%
	Significantly	6	4.0%
	Extremely	13	8.7%
Does your urinary incontinence affect your job or your normal daily activities such as exercises, shopping, etc.?	Not at all	67	44.7%
	A little	25	16.7%
	Slightly	18	12.0%
	Moderately	11	7.3%
	Significantly	10	6.7%
	Extremely	19	12.7%
Does urinary incontinence affect your driving?	Not at all	91	61.9%
	A little	15	10.2%
	Slightly	10	6.8%
	Moderately	13	8.8%
	Significantly	4	2.7%
	Extremely	14	9.5%
Does your bladder problem affect your ability to travel?	Not at all	84	56.4%
	A little	19	12.8%
	Slightly	13	8.7%
	Moderately	12	8.1%
	Significantly	8	5.4%
	Extremely	13	8.7%
Does your urinary incontinence affect your relationship with your partner?	Not at all	85	59.9%
	A little	15	10.6%
	Slightly	6	4.2%
	Moderately	10	7.0%
	Significantly	9	6.3%
	Extremely	17	12.0%
Does your urinary incontinence affect your sleep?	Not at all	78	52.3%
	A little	15	10.1%
	Slightly	15	10.1%
	Moderately	20	13.4%
	Significantly	7	4.7%
	Extremely	14	9.4%
Does your urinary incontinence make you feel worn out and tired, anxious, or nervous, or depressed?	Not at all	83	56.1%
	A little	19	12.8%
	Slightly	8	5.4%
	Moderately	16	10.8%
	Significantly	6	4.1%
	Extremely	16	10.8%
Does your urinary incontinence make you feel anxious or nervous?	Not at all	55	37.4%
	A little	25	17.0%
	Slightly	21	14.3%
	Moderately	18	12.2%
	Significantly	11	7.5%
	Extremely	17	11.6%
Does your urinary incontinence cause you to feel depressed?	Not at all	92	63.0%
	A little	16	11.0%
	Slightly	12	8.2%
	Moderately	7	4.8%
	Significantly	6	4.1%
	Extremely	13	8.9%

**Table 5:** Detailed analysis of the impact of urinary incontinence on various aspects of the participants’ daily lives.

Question	Response	N	%
How much water do you drink daily?	1–3 cups	61	36.5%
	4–6 cups	73	43.7%
	More than 6 cups	33	19.8%
How much coffee do you drink daily?	1–3 cups	80	54.8%
	4–6 cups	41	28.1%
	More than 6 cups	25	17.1%
How often do you urinate daily?	Less than 6 times	50	29.9%
	4–6 times	72	43.1%
	More than 6 times	45	26.9%
How often you get up at night to pass urine daily?	Once daily	89	61.8%
	Twice daily	35	24.3%
	Three times daily	20	13.9%
Have you experienced wetting the bed at night?	Yes	33	19.8%
	No	134	80.2%
Do you use pads as a solution for your condition?	Yes	71	47.0%
	No	80	53.0%
How often do you change your underclothes because of urine?	1 time daily	80	61.1%
	2 times daily	37	28.2%
	3 times daily	14	10.7%
Does wearing tight clothes affect your urinary incontinence?	Not at all	96	64.4%
	A little	38	25.5%
	Moderately	10	6.7%
	A lot	5	3.4%
Does your menstruation cycle make your condition worse?	Yes	44	29.1%
	No	107	70.9%
Does cold weather make your condition worse?	Yes	88	53.3%
	No	77	46.7%
Have you been physically abused and is the condition related to it?	Yes	8	5.3%
	No	142	94.7%
Do you have any fear or trauma, such as clown, animals, and aerophobia?	Yes	14	8.4%
	No	153	91.6%
Did the urinary incontinence start after you had anxiety?	Yes	39	23.6%
	No	126	76.4%

## Data Availability

The datasets presented in this article are not readily available due to technical/administrative limitations. Requests to access the datasets should be directed to Dr. Basim Alsaywid.
